# Allergic Interstitial Nephritis Manifesting as a Striated Nephrogram

**DOI:** 10.1155/2015/250530

**Published:** 2015-11-18

**Authors:** Irfan Moinuddin, Erika Bracamonte, Bijin Thajudeen, Amy Sussman, Machaiah Madhrira, James Costello

**Affiliations:** ^1^Division of Nephrology, University of Arizona Medical Center, Tucson, AZ 85724, USA; ^2^Department of Pathology, University of Arizona Medical Center, Tucson, AZ 85724, USA; ^3^Department of Medical Imaging, University of Arizona Medical Center, Tucson, AZ 85724, USA

## Abstract

Allergic interstitial nephritis (AIN) is an underdiagnosed cause of acute kidney injury (AKI). Guidelines suggest that AIN should be suspected in a patient who presents with an elevated serum creatinine and a urinalysis that shows white cells, white cell casts, or eosinophiluria. Drug-induced AIN is suspected if AKI is temporally related to the initiation of a new drug. However, patients with bland sediment and normal urinalysis can also have AIN. Currently, a definitive diagnosis of AIN is made by renal biopsy which is invasive and fraught with risks such as bleeding, infection, and hematoma. Additionally, it is frequently unclear when a kidney biopsy should be undertaken. We describe a biopsy proven case of allergic interstitial nephritis which manifested on contrast enhanced Magnetic Resonance Imaging (MRI) as a striated nephrogram. Newer and more stable macrocyclic gadolinium contrast agents have a well-demonstrated safety profile. Additionally, in the presentation of AKI, gadolinium contrast agents are safe to administer in patients who demonstrate good urine output and a downtrending creatinine. We propose that the differential for a striated nephrogram may include AIN. In cases in which the suspicion for AIN is high, this diagnostic consideration may be further characterized by contrast enhanced MRI.

## 1. Introduction

MR angiography has a role in evaluating patients with suspected renovascular hypertension and renal vein thrombosis and MRI is useful in the evaluation of renal masses, including suspected or confirmed renal cell carcinoma. MRI is especially useful for distinguishing and characterizing complex solid and cystic masses [1, 2].

The MR nephrogram, defined as the imaging of opacified renal parenchyma following the administration of contrast material, can provide indispensable insight into the dynamic function of the kidney. Receiving approximately 25% of cardiac output, the kidneys improve avidly following contrast agent administration. The Computed Tomography (CT) and Magnetic Resonance Imaging (MRI) nephrograms are analogous imaging descriptions of the enhancement pattern of the renal cortex and medulla. Any process that elicits interstitial inflammation or edema can produce a variant of the CT or MR nephrogram, specifically called a striated nephrogram. Such processes as pyelonephritis, renal contusion, and tubular obstruction can produce a striated nephrogram, characterized by linear bands of alternating contrast enhancement oriented parallel to the axis of the tubules and the collecting ducts. There is currently no available noninvasive test to substantiate the diagnosis of AIN. If clinical suspicion of AIN is high, the observation of a CT or MR striated nephrogram can provide valuable information to help guide the clinicians towards a diagnosis.

Early renal enhancement on CT and MRI is termed the corticomedullary phase, an imaging stage when the peripheral renal cortex enhances more than the central renal medulla. The corticomedullary phase is followed by the nephrographic phase in which the renal cortex and medulla improve to roughly the same degree. Finally, in the excretory phase, a functioning kidney excretes the administered contrast material [3, 4].

Global absence of the nephrogram highlights renal ischemia [5, 6]. Segmental absence may be due to renal space-occupying processes, hemodynamic changes in peripheral branches of the renal artery, or small vessel disease such as vasculitides [7, 8]. In the setting of renovascular compromise, the rim pattern represents preserved subcapsular perfusion by collateral flow [9, 10]. Temporal delay in the progression of the nephrogram may be attributed to a unilateral decrease in renal blood flow [11]. A persistent nephrogram may be due to systemic hypotension or acute tubular necrosis [12].

A striated nephrogram is characterized by linear bands of contrast enhancement oriented parallel to the axis of the tubules and collecting ducts. Unilateral striated nephrograms have been associated with ureteric obstruction, acute pyelonephritis [13], renal contusion [14], and renal vein thrombosis. Bilateral striated nephrograms have been demonstrated in acute pyelonephritis [13], tubular obstruction [15], hypotension [12], and autosomal recessive polycystic kidney disease [16].

One of the most important imaging findings on MR is the direct identification of inflammation using T2 fat-saturated imaging. T2 fat-saturated sequences remove the contribution of the fat signal, leaving a description of contributing water signal. This technique details inflammation and is instrumental in the diagnosis of other inflammatory etiologies of the abdomen including acute cholecystitis, pancreatitis, and enteritis [17, 18].

There is a paucity of research concerning the clinical relevance of the striated nephrogram. Even reports of the appearance of the striated nephrogram in cases of pyelonephritis, renal contusion, and tubular obstruction are few and far between. This is the first report of acute allergic interstitial nephritis manifesting on gadolinium-enhanced MRI as a striated nephrogram.

## 2. Materials/Methods and Results

A 28-year-old male engineering graduate student with no significant past medical history presented with acute renal failure. He was in his usual state of good health when he went swimming in a pool and overexerted himself. Shortly after swimming, he developed a dull, bilateral, nonradiating flank pain. The emergency room physician attributed the pain to muscle spasm or muscle strain and advised him to take ibuprofen. Patient took 200 mg ibuprofen every 8 hours for 24 hours without relief, prompting a visit to his primary care physician, who advised him to stop taking the ibuprofen. Routine labs revealed a creatinine of 671 micromol/L, and the patient was admitted for further evaluation.

In the inpatient setting, the renal ultrasound was normal. CBC showed a white blood cell count of 7500/microliter with 2.1% eosinophils. Urinalysis revealed mild proteinuria and mild hematuria: specific gravity 1.004/pH 6.0/protein 100/RBC 2/WBC 4. The urine protein to creatinine ratio was 444 mg/g. With unexplained renal failure, the patient underwent a prompt renal biopsy. With a mildly elevated LDH of 4.34 microkat/L and concern for renal infarct, an MRI of the abdomen with and without gadolinium contrast was ordered to better characterize the renal cortex and medulla. Complements C3 and C4 were normal at 0.145 g/L (normal: 0.8–1.6 g/L) and 0.047 g/L (normal: 0.015–0.053 g/L), respectively. Cytoplasmic anti-neutrophil cytoplasmic antibodies (C-ANCA), perinuclear anti-neutrophil cytoplasmic antibodies (P-ANCA), and anti-nuclear antibodies (ANA) were all negative.

**Figure 1:**
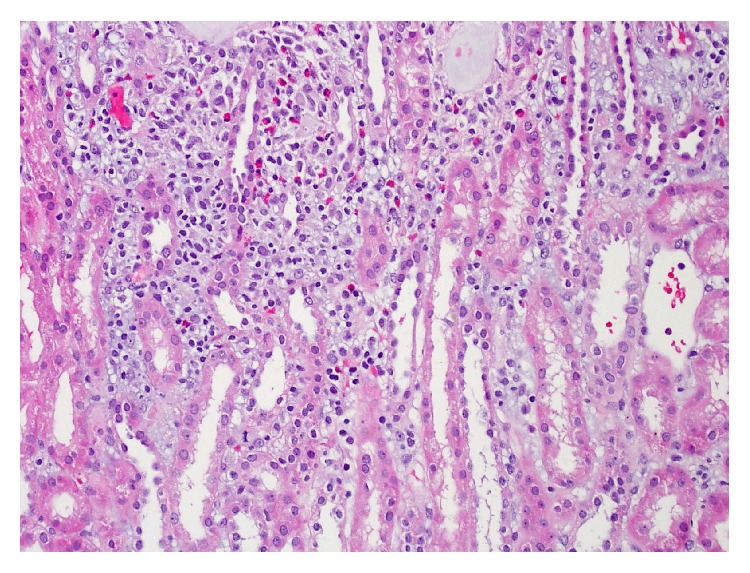
Renal cortex: the interstitium is expanded by edema and an inflammatory infiltrate containing lymphocytes, macrophages, and numerous eosinophils. H&E (200x magnification).

The kidney biopsy contained up to 23 viable glomeruli per level section with normal histologic appearance (no signs of inflammation, proliferation, segmental sclerosis, or other specific abnormalities). The interstitium was expanded by patchy edema and a mild to moderate chronic inflammatory infiltrate containing activated lymphocytes, histiocytes, and focal aggregates of eosinophils. There was no interstitial fibrosis or tubular atrophy. Muscular arteries and arterioles had no arteriosclerosis and showed no evidence of vasculitis. Immunofluorescence was performed; however, the sample was primarily renal medulla. Ultrastructural evaluation of the glomeruli was normal without evidence of immune deposits or podocyte abnormalities. The biopsy finding of active tubulointerstitial inflammation with eosinophils was most consistent with acute allergic interstitial nephritis (Figure 1).

**Figure 2:**
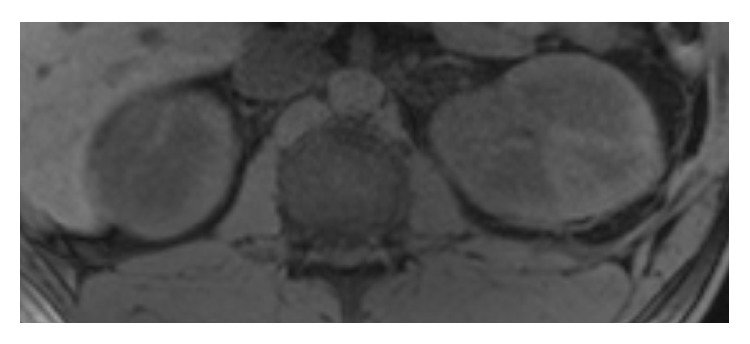
Axial fat-saturated T1w precontrast image of the kidneys: the unenhanced image does not provide dynamic information regarding the kidneys' uptake and excretion of contrast.

**Figure 3:**
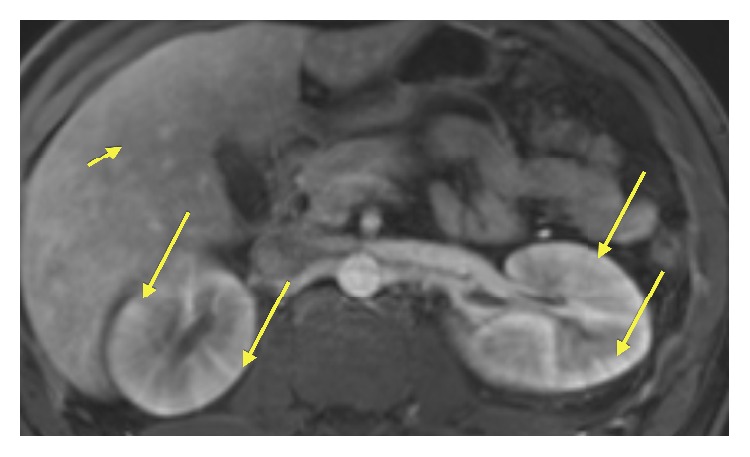
Axial fat-saturated T1w 20 second arterial phase contrast enhanced image: there is demonstration of heterogeneous enhancement of the renal cortex. Please note the “striated” appearance of the renal cortex (long arrows) which is also evident on 70-second venous phase and 180-second delayed venous phase imaging. Incidentally, the liver parenchyma also demonstrates heterogeneous enhancement (short arrow), consistent with inflammation.

**Figure 4:**
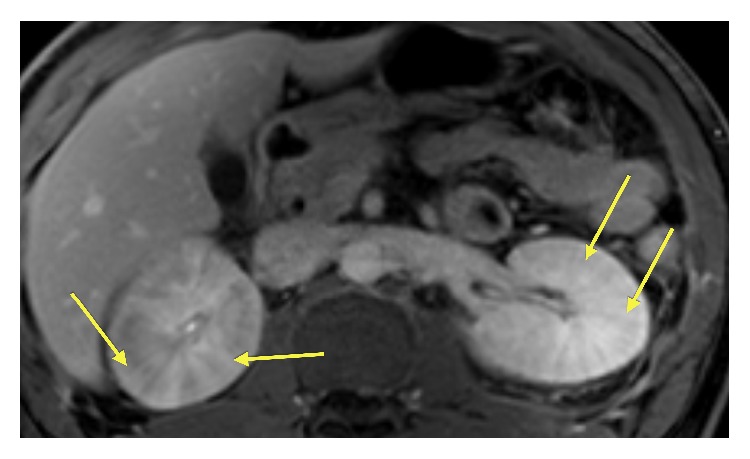
Axial fat-saturated T1w 180 second delayed venous phase contrast enhanced image: there is better conspicuity of the “striated” enhancement pattern (arrows) which is evident in both the renal cortex and the medulla. This finding contrasts with Figure 3 where the “striated” pattern is only evident with the renal cortex.

**Figure 5:**
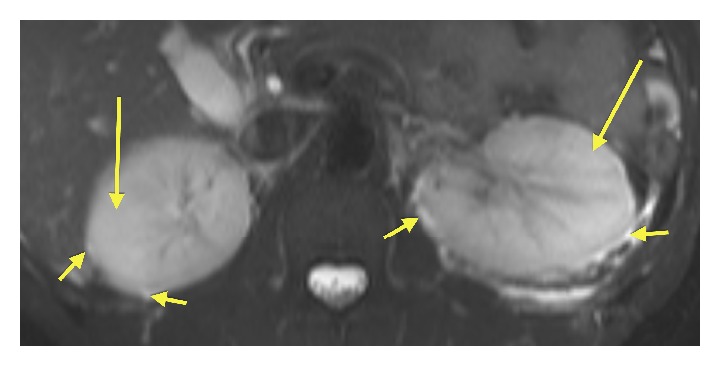
Axial fat-saturated T2w image of the kidneys: the image demonstrates high signal (long arrows) within the renal cortex. Additionally, there is fluid signal and inflammation identified in the perinephric space (short arrows).

The MRI of the abdomen demonstrated bilateral edematous kidneys with loss of the normal cortical medullary differentiation pattern. The unenhanced image does not provide dynamic information regarding the kidneys' uptake and excretion of contrast (Figure 2). Furthermore, the kidneys demonstrate a “striated nephrogram” appearance which is best visualized on contrast enhanced T1 fat-saturated imaging (Figure 3). This finding is best highlighted on 70-second portal venous phase imaging and 180 sec delayed phase imaging (Figure 4). The liver also demonstrated heterogeneous enhancement on T1 fat-saturated 20 sec arterial phase imaging. This finding is consistent with reactive inflammation of the liver (Figure 3). Axial T2-weighted fat-saturated images demonstrated increased inflammatory signal in the bilateral kidneys with the surrounding perinephric fluid (Figure 5).

Patient was diagnosed with AIN attributable to unknown causes and treated with oral prednisone for a short course of two weeks with subsequent decrease in serum creatinine to 55.7 micromol/L.

## 3. Discussion

The patient presented with a markedly elevated creatinine and acute renal failure. What were the possible contributing etiologies? Even sudden and complete loss of renal function would not raise the serum creatinine from normal to this level in only one day. It is far more likely that the renal injury causing AKI predated the creatinine measurement by at least 3 days or more. Thus, the AKI was not attributable to ibuprofen, particularly in a scenario of only three administered doses. Additionally, the patient reported no other outpatient medications. Within this context, the patient was diagnosed with AIN of unknown cause.

Although some degree of peripheral eosinophilia may be present in AIN, peripheral eosinophilia is not an absolute criterion for the diagnosis of AIN. Urine eosinophils (EOS) demonstrate a prolonged turnaround time with poor sensitivity and specificity, and they are no longer recommended in the evaluation of AIN. In one study, urine EOS were found in a variety of kidney diseases besides AIN. At the commonly used 1% urine eos cutoff, the test does not shift pretest probability of AIN in any direction. Even at a 5% cutoff, urine eos performed poorly in distinguishing AIN from acute tubular necrosis or other kidney diseases [19].

The biopsy showed patchy edema and focal aggregates of eosinophils. This is consistent with AIN. Most cases of acute interstitial nephritis are characterized by focal infiltrates that are globally present [20]. A diffuse interstitial inflammatory process is not a prerequisite for the diagnosis of AIN. The biopsy also showed one or two partially denuded tubules, also consistent with AIN and tubular damage [21, 22].

In acute tubulointerstitial inflammation or edema, the cellular inflammatory infiltrate selectively increases parenchymal pressure within the affected medullary or cortical rays. Following the administration of an imaging contrast agent, these areas initially demonstrate decreased enhancement. With delayed imaging, the same areas may demonstrate enhancement that is increased relative to that of adjacent normal tissue. This nephrographic reversal is caused by tubular stasis in which contrast enters slowly and becomes hyperconcentrated in collecting ducts obstructed by inflammatory cells [23]. The end result is a striated nephrogram characterized by linear bands of alternating contrast enhancement oriented parallel to the axis of the tubules and the collecting ducts.

In the setting of acute kidney injury, questions may arise when ordering a gadolinium contrast enhanced MR exam. For patients with an acute drop in glomerular filtration rate (GFR), heightened attention must be heeded, but as long as the patient continues to produce urine and the GFR has demonstrated progressive interval improvement over an interval of a few days, a contrast enhanced MR exam can be pursued [24]. Moreover, recent data documents that the risk of nephrogenic systemic fibrosis is minimal with the newer and far more stable macrocyclic MR contrast agents. In the case of patients with good urine output and without documented stage 4 or stage 5 chronic kidney disease, the data indicates no increased risk for nephrogenic systemic fibrosis [25].

This report constitutes the first report of allergic interstitial nephritis manifesting on MRI as a striated nephrogram. The implications of this study are that allergic interstitial nephritis must be considered in the differential diagnosis of the striated nephrogram. Currently, there is no noninvasive means of substantiating a diagnosis of acute allergic interstitial nephritis. Our experience suggests that MR imaging evaluation of suspected cases of acute interstitial nephritis warrants further investigation.
